# Long-term glycine propionyl-l-carnitine supplemention and paradoxical effects on repeated anaerobic sprint performance

**DOI:** 10.1186/1550-2783-7-35

**Published:** 2010-10-28

**Authors:** Patrick L Jacobs, Erica R Goldstein

**Affiliations:** 1 Department of Exercise Science, Florida Atlantic University, Boca Raton, FL 33431, USA

## Abstract

**Background:**

It has been demonstrated that acute GPLC supplementation produces enhanced anaerobic work capacity with reduced lactate production in resistance trained males. However, it is not known what effects chronic GPLC supplementation has on anaerobic performances or lactate clearance.

**Purpose:**

The purpose of this study was to examine the long-term effects of different dosages of GPLC supplementation on repeated high intensity stationary cycle sprint performance.

**Methods:**

Forty-five resistance trained men participated in a double-blind, controlled research study. All subjects completed two testing sessions, seven days apart, 90 minutes following oral ingestion of either 4.5 grams GPLC or 4.5 grams cellulose (PL), in randomized order. The exercise testing protocol consisted of five 10-second Wingate cycle sprints separated by 1-minute active recovery periods. Following completion of the second test session, the 45 subjects were randomly assigned to receive 1.5 g, 3.0 g, or 4.5 g GPLC per day for a 28 day period. Subjects completed a third test session following the four weeks of GPLC supplementation using the same testing protocol. Values of peak power (PP), mean power (MP) and percent decrement of power (DEC) were determined per bout and standardized relative to body mass. Heart rate (HR) and blood lactate (LAC) were measured prior to, during and following the five sprint bouts.

**Results:**

There were no significant effects of condition or significant interaction effects detected for PP and MP. However, results indicated that sprint bouts three, four and five produced 2 - 5% lower values of PP and 3 - 7% lower values of MP with GPLC at 3.0 or 4.5 g per day as compared to baseline values. Conversely, 1.5 g GPLC produced 3 - 6% higher values of PP and 2 -5% higher values of MP compared with PL baseline values. Values of DEC were significantly greater (15-20%) greater across the five sprint bouts with 3.0 g or 4.5 g GPLC, but the 1.5 g GPLC supplementation produced DEC values -5%, -3%, +4%, +5%, and +2% different from the baseline PL values. The 1.5 g group displayed a statistically significant 24% reduction in net lactate accumulation per unit power output (p < 0.05).

**Conclusions:**

The effects of GPLC supplementation on anaerobic work capacity and lactate accumulation appear to be dosage dependent. Four weeks of GPLC supplementation at 3.0 and 4.5 g/day resulted in reduced mean values of power output with greater rates of DEC compared with baseline while 1.5 g/day produced higher mean values of MP and PP with modest increases of DEC. Supplementation of 1.5 g/day also produced a significantly lower rate of lactate accumulation per unit power output compared with 3.0 and 4.5 g/day. In conclusion, GPLC appears to be a useful dietary supplement to enhance anaerobic work capacity and potentially sport performance, but apparently the dosage must be determined specific to the intensity and duration of exercise.

## Introduction

Exercise capacity is generally considered as the greatest amount of physical exertion that can be sustained at a given level of intensity. Success in endurance sports is related to an ability to continue with relatively high efforts for extended periods of time. In contrast, most team sports involve intermittent bouts of high intensity exertion with limited recovery intervals. A number of strategies are commonly utilized to increase exercise capacity as a means of enhancing sport performance. These include various approaches to training and conditioning as well as nutritional strategies to improve peak exercise capacity as well as exercise efficiency.

While numerous factors underlie exercise capacity, a primary consideration is that of energy demand versus energy supply. The intensity of exercise corresponds- to a great degree- to the specific energy demands of the activity. The capacity to perform at a given intensity of effort is limited by the localized energy supplies and the ability to replenish those energy stores as exercise continues. In conjunction with the increased metabolic demand for energy during exercise, there is increased blood flow to the exercising muscles [1]. During exercise, the vasculature system is the sole means to deliver energy replenishment as well as to remove metabolites that may limit ongoing efforts. A close pairing of exercise intensity and local blood flow suggests that potential strategies capable of increasing blood flow to exercising muscles may enhance maximal work capacity and/or increase resistance to localized muscle fatigue during ongoing exercise at submaximal intensities.

The process of increasing blood flow to exercising musculature involves shunting of blood from non-active tissues to working muscle. As physical exercise increases in intensity, there are a number of mechanisms involved in the vasodilation of the arterioles and the pre-capillary sphincters [2]. These vasodilatory mechanisms are diverse but share two distinct characteristics in that the activity of each of the differing mechanisms increases in direct response to increasing intensities of exercise and those mechanisms all initiate the synthesis of nitric oxide (NO). Nitric oxide is the endothelial factor responsible for relaxation of smooth musculature surrounding the arterials and the pre-capillary sphincters thereby producing vasodilation and increased blood flow into the capillary bed of the exercising muscle tissue. Since its identification approximately twenty years ago, various research studies and subsequent sports nutrition products have emerged in an effort to manipulate levels of NO in order to enhance exercise performance. This quest has resulted in a sizable nutritional supplement market, primarily composed of arginine based products. While arginine is the precursor of NO, there is no scientific evidence to support such an approach. In fact, all published studies in this area indicate that oral administration of arginine in dosages tolerated by the gastrointestinal system are not effective in producing endothelium-dependent vasodilation or in elevating NO levels [3-5].

It has been demonstrated that short term administration of an oral carnitine compound, glycine propionyl-L-carnitine (GPLC), produces significantly elevated levels of nitric oxide metabolites at rest in both sedentary and trained persons [6,7]. Increased nitric oxide activity has also been demonstrated in resistance trained persons with reactive hyperaemia testing, an assessment used in clinical settings that, to some degree, simulates the physical stresses encountered during very intense exercise such as resistance training [7]. These studies are the first to document the effectiveness of an oral nutritional supplement to directly affect NO synthesis.

It has also been recently shown that acute GPLC supplementation (4.5 g) enhances anaerobic work capacity with reduced lactate production in resistance trained males [8]. However, little is known regarding the effects chronic GPLC supplementation has on exercise performance in trained persons. It was the purpose of the present investigation to examine the effects of 28 days of varying GPLC dosing on anaerobic work capacity and lactate accumulation.

## Methods

### Research Participants

Forty-five male resistance trained individuals volunteered to participate in this double-blind investigation. Study inclusion criteria limited research subjects to males between the ages of 18 and 35 years, who reported participation in at least two weekly resistance training sessions over the six-month period immediately prior to the start of the study. Exclusionary criteria included any reported history of significant cardiorespiratory complications or recent lower extremity musculoskeletal injury that might limit high intensity exercise efforts. Subjects provided written informed consent after verbal explanation of all study procedures, in accordance with the Institutional Medical Sciences Subcommittee for the Protection of Human Subjects.

### Study Design

All subjects were asked to complete three testing sessions. The first two test sessions were performed one week apart with the third trial scheduled 28 days later. The first two tests were performed 90 minutes following oral ingestion of either 4.5 grams GPLC or 4.5 grams cellulose (PL), in randomized order. The exercise testing protocol consisted of five 10-second Wingate cycle sprints separated by 1-minute active recovery periods. The findings of this acute study, presented in a previous publication, reported significantly increased power output with reduced lactate accumulations with acute GPLC supplementation (Jacobs, 2009).

The present investigation is a continuation of our acute study of GPLC in which a randomized blocks design was implemented to examine the long-term effects of varying dosages of GPLC. All of the present subjects completed acute testing with GPLC and PL in order to provide a consistent subject test exposure for the present investigation. The PL condition served as the control/baseline condition for the present study. Pilot testing had indicated that the majority of persons could correctly identify to GPLC condition compared with placebo. As it is well established that subject compliance and retention are significantly reduced when a placebo condition is identified, the present design was utilized in which the placebo condition of the first two assessments served as the baseline condition, each subject serving as their own control. Subjects were matched by body mass and then randomly assigned to one of three study groups, with one group receiving 1.5 grams per day of GPLC, one group receiving 3.0 grams GPLC per day, and the final group receiving a daily dosage of 4.5 grams of GPLC. (See Supplementation Protocol Section).

During the one month supplementation period, subjects were directed to continue with their own individual training and nutritional programs. Seven day exercise logs and three day dietary recall logs were completed by all subjects to provide verification of the consistency of training and diet. These exercise and dietary records were submitted for the weeks prior to baseline and post supplementation testing. The exercise logs provided information regarding exercise volume (sets, reps) of resistance training categorized to upper extremity, lower extremity, or structural movements. The dietary intake logs were examined using ESHA Food Processor SQL dietary analysis software (ESHA Research, Salem, OR).

All subjects were scheduled for a third cycle sprint session following the 28 days of supplementation. As with the prior assessments, subjects were asked to report for testing in the morning following 12 hr without food and to not participate in heavy exercise during the 24 hr period before testing. On test day, the subjects were provided with the same dosing as they had taken during the 28 day supplementation period. All subjects sat quietly for 90 minutes after taking the supplement before participating in the cycle sprint testing.

### Supplementation Protocol

Subjects were matched by body mass and then randomly assigned to one of three study groups, each group receiving 28 days of GPLC supplementation in one of three dosages (1.5 g/d, 3.0 g/d, 4.5 g/d). In a double blind fashion, each subject was provided with 28 packets consisting of six capsules per day. The daily packets included six 750 mg capsules provided by Jarrow Formulas (Los Angeles, CA). The respective daily dosage was established by the appropriate combination of 750 mg GPLC capsules and 750 mg capsules of cellulose (the GPLC and cellulose capsules were visually identical). For example, the daily packets of the 1.5 g/d group were comprised of two GPLC capsules and four cellulose capsules while the 3.0 g/d group received four GPLC and two cellulose capsules and the 4.5 g/d group was provided with six GPLC capsules. Participants were directed to take their six capsule daily supplements approximately 90 minutes prior to exercise on training days and to take the six capsules with breakfast on other days. The GPLC used in this study was the USP grade nutritional product, GlycoCarn™ (Sigma Ta Health Sciences, S.p.A., Rome, Italy), a molecularly bonded form of glycine and propionyl-L-carnitine.

### Assessment Protocol

The testing protocol used in the present investigation is consistent with that previously described by these investigators (Jacobs, 2009). Briefly, this testing protocol included five high intensity stationary cycle sprints, each sprint 10-seconds in duration with 1-minute active recovery periods. Sprints were performed with a Monarch 894E leg ergometer (Monarch, Varberb, Sweden) with the external applied resistance equivalent to 7.5% of each subject's body mass. Ten minutes of unloaded pedalling at 60 RPM was performed as a warm-up prior to the sprint testing. The 1-minute recovery periods were active with unloaded pedalling with cadence fixed at 60 RPM.

**Anaerobic power output **was measured using the SMI OptoSensor 2000 (Sports Medicine Industries, Inc., St. Cloud, Minn). Power output variables included peak power (PP) which was determined as the power output established during the first 5 seconds of each ten second sprint; and mean power (MP) which was the power output measured during the full ten seconds of each sprint. The third power output variable was a power decrement (DEC) which was calculated as the difference in power output between the first 5 seconds and the second five seconds of each sprint, as expressed as a percentage of the first 5 second period.

**Heart rate **(HR) was determined using a Polar HR monitoring system with HR values assessed at rest, during the final five seconds of each sprint bout, as well as four and fourteen minutes after the final sprint bout.

**Blood lactate levels **(LAC) were assessed using the Accutrend^® ^lactate analyzer (Sports Resource Group, Inc., Pleasantville, NY). Calibration procedures were performed prior to each testing session using standard control solutions. Blood lactate levels were determined at rest as well as four and fourteen minutes post exercise. Net lactate accumulation per unit power output was calculated as (LAC_14_-LAC_rest_)·(MP_ave_)^-1 ^.

**Thigh girth **of the dominant leg was measured using a Gulick tape at 15 mm superior to the patella while in a standing position with weight shifted onto the non-dominant leg. Thigh girth measurements were taken at rest and four minutes after the final sprint bout.

### Statistical Analyses

A repeated measures general linear model was used to examine for differences in outcome measures between groups (1.5 g/d, 1 g/d, 4.5 g/d), conditions (pre- and post-GPLC) and across time. Measures of power output (PP, MP, DEC) were determined across time during each of the five successive sprint bouts. Values of HR were established at rest, during the final five seconds of each sprint, as well as four and fourteen minutes following the last sprint. The across time measures of LAC were taken at rest as well as four and fourteen minutes post exercise while thigh girth was assessed at rest and four minutes after the fifth sprint. In cases where significant main effects or interactions were observed, single degree of free contrasts were performed to determine specific effects without adjustment of the acceptable level of significance. Net lactate accumulation was calculated as the difference between lactate measurements 14 min post exercise and resting values divided by the average MP values of the five sprints. In all cases, p-values less than 0.05 were accepted to determine statistical significance. All analyses were performed using PASW, Version 17.

## Results

### Research Participants

Of the 45 participants enrolled for this study, 38 individuals completed all study assessments. All statistical analyses were based on the data derived from participants who completed all requisite testing sessions. The total subject pool consisted of 13 subjects from the 1.5 g/d group, 11 subjects from the 3.0 g/d group and 14 subjects from the 4.5 g/d group, respectively. The seven participants who did not complete the study testing included three individuals that developed musculoskeletal injuries from other activities (intramural sports), two that did not maintain consistent levels of exercise training, and two that declined to participate in the final sprint testing session. Subject demographics are provided by group in Table 1. There were no significant differences between groups in demographic factors.

**Table 1:** Subject Demographics

	1.5 g/d	3.0 g/d	4.5 g/d
Age (yrs)	25.5 ± 6.4	24.8 ± 4.9	23.6 ± 3.4
Body Mass (kg)	89.6 ± 14.3	84.2 ± 11.2	84.3 ± 17.2
Height (cm)	179.0 ± 4.4	178.7 ± 7.6	173.5 ± 5.7

### Dietary Log Data

Table 2 provides macronutrient intake values of each of the three supplementation groups, for the one-week period prior to initial and post-treatment testing. Analyses indicated that there were no significant differences between groups at baseline or at post-testing in the dietary intake of carbohydrates, fats, or protein or in the values of total calories ingested. Nor were there any significant differences detected within groups between the initial and post-treatment assessments.

**Table 2:** Nutritional Recall Information

		1.5 g/d	3.0 g/d	4.5 g/d
**Carbohydrates (g)**	**Baseline**	**210.3 ± 91.5**	**254.5 ± 149.5**	**238.2 ± 115.1**
	**4 weeks**	**257.0 ± 143.6**	**254.4 ± 162.2**	**242.1 ± 117.9**

**Fats (g)**	Baseline	76.5 ± 24.2	62.1 ± 25.2	76.5 ± 38.4
	4 weeks	58.0 ± 16.4	65.0 ± 29.2	73.4 ± 43.1

**Protein (g)**	**Baseline**	**190.3 ± 82.6**	**178.3 ± 92.5**	**165.8 ± 76.4**
	**4 weeks**	**197.6 ± 76.0**	**163.1 ± 109.5**	**178.4 ± 78.6**

**Total Calories (kcal/day)**	Baseline	2322.1 ± 528.0	2229.5 ± 717.2	2317.8 ± 661.2
	4 weeks	2264.9 ± 574.1	2160.8 ± 901.1	2418.2 ± 760.3

### Exercise Log Data

Participants were asked to complete a log of all resistance training exercise performed during the week prior to initial testing and last week of the four week study. These logs included the number of sets per exercise, with exercises classified by investigators as either upper or lower extremity and also as either single-joint or multi-joint movements. The training volume values are presented in Table 3. Analyses revealed no significant differences between study groups in the number of sets or repetitions regardless of exercise categories.

**Table 3:** Resistance Training Log Data

		1.5 g/d		3.0 g/d		4.5 g/d	
		Baseline	4 weeks	Baseline	4 weeks	Baseline	4 weeks
**Upper Extremity Compound Exercises**	**Sets**	**40.6 ± 16.8**	**39.7 ± 19.3**	**40.8 ± 16.1**	**46.0 ± 24.6**	**42.8 ± 21.1**	**34.4 ± 15.0**
	**Reps**	**469.3 ± 347.1**	**379.2 ± 191.7**	**398.9 ± 204.1**	**413.2 ± 189.1**	**521.9 ± 421**	**341.8 ± 210.5**

**Upper Extremity Single Joint Exercises**	Sets	35.9 ± 19.1	35.5 ± 25.9	34.5 ± 23.1	33.8 ± 22.3	42.0 ± 22.8	41.2 ± 30.5
	Reps	453.8 ± 287.4	391.2 ± 352.5	380.8 ± 281.4	333.9 ± 192.6	541.4 ± 308.1	448.2 ± 429.4

**Lower Extremity Compound Exercises**	**Sets**	**9.3 ± 7.8**	**13.9 ± 12.7**	**10.7 ± 9.2**	**14.6 ± 17.7**	**7.2 ± 6.3**	**12.9 ± 8.1**
	**Reps**	**106.8 ± 135.5**	**141.0 ± 168.8**	**113.0 ± 103.3**	**153.7 ± 316.7**	**89.7 ± 153.0**	**113.9 ± 81.1**

**Lower Extremity Single Joint Exercises**	Sets	8.2 ± 8.6	6.9 ± 6.8	8.2 ± 7.5	7.4 ± 4.4	8.4 ± 9.5	7.4 ± 8.1
	Reps	131.7 ± 251.0	73.4 ± 73.2	93.7 ± 88.4	82.1 ± 67.5	153.6 ± 316.8	67.1 ± 78.3

### Power Output

Analyses indicated statistically significant main effects for time (bout order) for PP, MP, and DEC (p's < 0.001). In general, values of PP and MP tended to decrease in value with ongoing sprint bouts while DEC tended to increase. There were no significant differences detected among the three study groups (1.5 g/d, 3.0 g/d, 4.5 g/d) in baseline power values.

#### Peak Power

**Figure 1:**
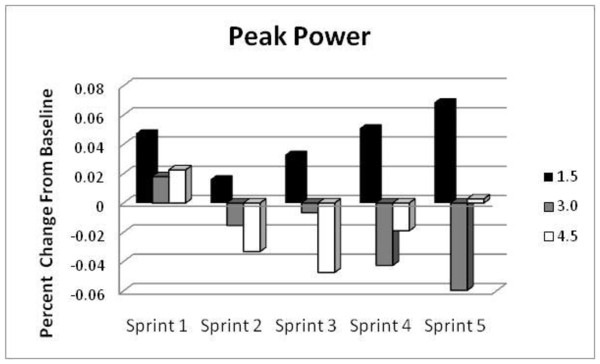
**Percent change of Peak Power (PP) from baseline determined during repeated cycling sprints in the 1.5 g/d group (black columns), in the 3.0 g/d group (gray columns) and in the 4.5 g/d group (white columns)**.

Changes in PP from baseline with supplementation across the five sprints are graphically presented in Figure 1. Values of PP were 4.7%, 1.6%, 3.3%, 5.1%, and 6.8% higher with the 1.5 g/d dosage compared with baseline values. Conversely, the 3.0 g/d group displayed 4.3% and 6.0% lower values of PP with the 4^th ^and 5^th ^sprint and the PP was up to 4.7% lower with the 4.5 g/d dosage. Despite the differences between mean group PP values, there were no statistically significant main effects of GPLC or interactions.

#### Mean Power

**Figure 2:**
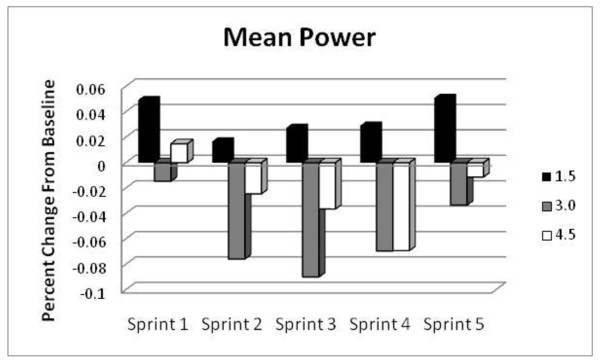
**Percent change of Mean Power (MP) from baseline determined during repeated cycling sprints in the 1.5 g/d group (black columns), in the 3.0 g/d group (gray columns) and in the 4.5 g/d group (white columns)**.

Figure 2 provides a visual depiction of the mean changes in MP with treatment for the three groups. The 3.0 g/d group produced considerably less MP on all five sprints (-1.5%, - 7.6%, -9.0%, -7.0, -3.3) and the 4.5 g/d group had lower values of MP on sprints two through five (-2.5%, -3.6%, -6.9%, -1.1). In contrast, greater MP was reached on all bouts with the 1.5 g/d dosage with gains across the five sprints of +4.9%, +1.7%, +2.7%, +2.9%, and +5.1% compared with baseline. No statistically significant effects of treatment or factor interactions were detected.

#### Power Decrement

**Figure 3:**
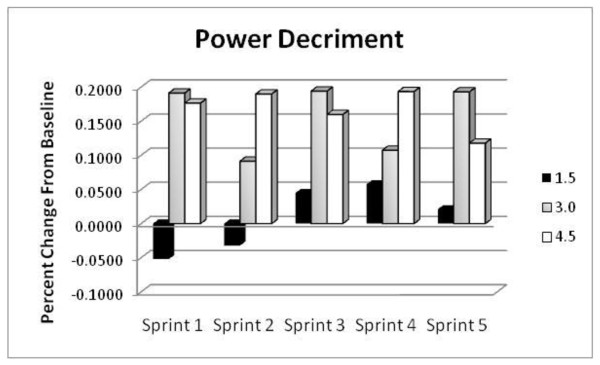
**Percent change in the decrement in power output (DEC) from baseline determined during repeated cycling sprints in the 1.5 g/d group (black columns), in the 3.0 g/d group (gray columns) and in the 4.5 g/d group (white columns)**.

In addition to the significant effect of time previously mentioned, DEC values were also observed to be significantly affected by condition (pre- and post-GPLC) and by a condition x group interaction (p < 0.05). These statistics suggest that the rate of power decrement across the five sprint bouts changed from baseline differentially among the three supplement levels. Figure 3 provides an illustration of the contrasting changes in DEC between groups. Values of DEC were appreciably greater with the 3.0 g/d dosage (+19.1%, +9.1%, +19.4%, +10.7%, +19.3%) and with the 4.5 d/g intake (+17.6%, +19.0%, +16.0%, +19.3%, + 11.8%). The 1.5 g/d group displayed lower values of DEC on the first two sprints (-5.2%, -3.22%) with DEC on sprints three through five 2 - 5% higher than initial values. In general, the 3.0 and 4.5 g/d groups exhibited dramatically greater rates of DEC compared with baseline while the 1.5 g/d dosage resulted in greater resistance to fatigue on sprints 1 and 2 with more modest changes in DEC with sprints 3 -5.

### Lactate

Lactate values at baseline, 4 and 14 min post exercise in each of the three supplementation groups are provided in Table 4. LAC values were significantly different across time in all groups (p < 0.05) with greater values post-exercise (4 and 14 min) compared with baseline values. The general pattern of reduced lactate accumulation with GPLC is apparent to some degree in the three study groups, but only the 1.5 g/d group displayed a strong trend (p = 0.07) for statistically significant reduction in absolute blood lactate levels at 14 min post sprints. Net lactate accumulation per unit power output was calculated as (LAC_14_-LAC_rest_)·(MP_ave_)^-1 ^with values only differing with GPLC in the 1.5 g/d group. The 1.5 g/d GPLC supplementation group exhibited a 24.1% reduction in net lactate per watt (1.44 to 1.09 mmol^.^watt^-1^) (p < 0.05). The 3.0 g/d group actually produced 27.0% more lactate per unit watt (.80 to 1.02 mmol^.^watt^-1^) and the 4.5 g/d group displayed a non-significant 11.6% reduction (1.24 to 1.09 mmol^.^watt^-1^). The change in net lactate accumulation per unit power output of the 1.5 g/d group was significantly greater than the changes exhibited by the other two groups (p < 0.05).

**Table 4:** Lactate Measurements (mmol·L^-1^)

		Resting	4-min post	14- min post
**1.5 g/d**	**Baseline**	**1.3 ± 0.4**	**11.3 ± 4.0**	**11.8 ± 2.5**
	**4 weeks**	**1.5 ± 0.4**	**11.0 ± 3.3**	**9.4 ± 4.4**

**3.0 g/d**	Baseline	1.8 ± 0.7	11.6 ± 3.4	8.2 ± 3.0
	4 weeks	1.6 ± 0.4	10.5 ± 4.4	9.4 ± 4.1

**4.5 g/d**	**Baseline**	**1.8 ± 0.4**	**12.2 ± 3.0**	**11.9 ± 4.2**
	**4 weeks**	**1.6 ± 0.6**	**11.5 ± 3.7**	**9.6 ± 3.6**

### Heart Rate

There were no significant main effects or significant interactions detected in values of HR at rest, during or following the five sprints. The mean HR responses were similar in the three study groups at rest (approximately 61-63 bpm) and in response to the sprint bouts with mean HRs increasing from 150-155 bpm to approximately 170 bpm from the first to fifth sprint bout. Recovery HR values did not differ appreciably between group with HR values of 125-130 and 110-125 bpm at four and 14 minutes following sprinting, respectively.

### Thigh Girth

Analyses revealed no significant effects of GPLC in any dosage or interactions in regard to thigh circumferential measurements. There was a significant time effect as the post-exercise assessment produced greater thigh girth measurements with exercise across all study participants. However, while there were no statistically significant interaction effects with the supplementation level (groups) it is interesting to note that while the 3.0 and 4.5 g/d groups displayed similar increases in mean thigh girth with treatment (3.0 g/d: 1.7 to 2.2 cm; 4.5 g/d: 1.7 to 2.0 cm) the 1.5 g/d study group displayed acute increases of thigh girth of 1.3 cm both at baseline testing and after four-weeks of supplementation.

## Discussion

Findings of the present investigation suggest that increasing daily intake of GPLC has somewhat paradoxical influences on the performance of repeated high intensity cycle sprints. These authors have previously reported that GPLC may produce acute enhancement of anaerobic power output during repeated cycle sprints [8]. Based on those results, it was speculated that long-term supplementation would, in general, provide further performance enhancements with those improvements related directly to the greater duration of supplementation and to the daily GPLC intake. However, these current findings indicate that long-term GPLC supplementation at the higher dosages examined (3.0 and 4.5 g/d) did not result in greater values of power output but rather lower mean values of PP and MP. In contrast, the lower intake group (1.5 g/d) exhibited mean values of PP and MP greater than baseline across the five sprints. Those increases in power output were similar to those previously reported with acute intake of 4.5 g GPLC.

The results of this study are not sufficient to definitively explain the apparent decline in sprint performance with higher GPLC intake. However, examination of the mechanisms of action may allow useful supposition. Potential mechanisms involved in the observed acute performance improvements include the unique vasodilatory actions of GPLC as well as supply of an energy source in the form of the propionyl group. An increased cellular supply of carnitine may also provide anaerobic buffering thereby reducing lactate production and enabling greater resistance to fatigue with high intensity exercise. It is likely that blood serum and tissue concentration levels of carnitine and propionate increase over time to some point of saturation. It is recommended that future investigations examine the time by dosage dynamics involved in GPLC supplementation.

The mechanisms involved in acute enhancement of power output and reduced lactate accumulation are possibly (in higher intake levels) also responsible for the reduced mean values of power seen with long-term intake. These authors suggest that it is unlikely that greater levels of propionate or carnitine in the blood stream or muscle tissue would reduce the production of power during the repeated sprints. However, it appears quite probable that the vasodilatory effects of GPLC surpassed a beneficial magnitude in the 3.0 and 4.5 g/d groups. A post-hoc examination of participant statements regarding their condition following the final testing session revealed that 13 of the 38 individuals completing the study complained that discomfort associated with leg pump limited their sprinting performance. These 13 included five of the 12 individuals in the 3.0 g/d group, and seven of the 14 participants in the 4.5 g/d group but only one individual in the 1.5 g/d group reported leg pump as a limiting factor. While not statistically significant, the 3.0 and 4.5 g/d groups displayed greater mean increases in thigh girth with sprinting compared with baseline while the 1.5 g/d group exhibited the same relative leg pump. Thus, while the results of this study cannot definitively explain the lack of power output enhancement with long-term intake of GPLC, the limited information available suggests that excessive localized muscle pumping is involved.

With increasing intensity of exercise, there is proportional increase in local blood flow of the exercising musculature. Vasodilation provides up to 25 -50 times resting levels of local blood flow by means of relaxation of the smooth arterial musculature and of the sphincter allowing flow into the capillary bed [9]. The process of vasodilation is closely associated with NO as this short-lived, reactive nitrogen molecule is responsible for regulation of vascular muscle tone [10]. Since it was determined that NO has a vital role in the control of blood flow, scientists have speculated on the effects increased levels would have on cardiovascular functioning in particular and exercise performances in general. However, this question has remained a matter of supposition as no nutritional supplementation has proven capable of influencing NO synthesis, until recently.

The only food supplement shown to directly affect the production of NO is GPLC. It has been shown that 28 d GPLC at 4.5 g/d produces significantly elevated levels of nitrites and nitrates [6,7]. Acute supplementation at 4.5 g resulted in significant improvements in cycling power output [8], but in the current study long-term supplementation of GPLC at that daily intake was not associated with power enhancement. Rather after 28 d GPLC at 4.5 g/d there was a significantly greater rate of power decline within individual sprints with reduced mean power output. In contrast, 28 d at a lower dosage, 1.5 g/d, provided increased mean values of power similar to those exhibited acutely with 4.5 g. The increases in NO reported after 28 d GPLC at 4.5 g/d are apparently associated with the extreme leg pump that limited cycling power in the present study. Similarly, with 4.5 g/d there was a significant reduction in net lactate accumulation per unit power acutely - with like reductions also observed after 28 d at 1.5 g/d, but not but not after 28 d at 4.5 g/d. Apparently, the long-term effects of GPLC are related to the timed effects of different individual mechanisms. The vasodilatory effects are certainly directly related to NO levels while the increased power output may be related to increased cellular supply of the propionate unit which when converted to succinate provides an anaplerotic energy substrate. Greater carnitine supply may be responsible for the reduced lactate accumulation due to buffering of the Coenzyme A pool thereby reducing the rate of fatigue and enabling a higher rate of power output. It would appear that both the vasodilatory effects and power output enhancement effects increased in magnitude over the 28 d period of the present study.

The present study is limited by several factors including a modest sample size which restricted the statistical analyses. Some variability within groups could be associated with the lack of control of the study supplement. Study participants were provide with 28 days of GPLC in the respective group levels and directed to take six capsules daily. However, there were no means available to ensure daily intake of the respective supplements. This investigation applied three absolute dosage levels (1.5, 3.0, 4.5 g/d) in all research participants. The absolute dosing regardless of body mass likely increased the variability of response within supplementation groups thereby limiting the findings of the present study. It is recommended that future investigations examine GPLC dosing relative to body mass.

Regardless of these potential limitations, the total subject pool in this study did not display the same main effects for enhancement of power output with reduced lactate accumulation as had been observed with acute supplementation. While the lower intake group (1.5 g/d) did display improvements in mean values of power output with significantly lower net lactate accumulation per unit power output, the higher intake groups (3.0 and 4.5 g/d) actually produced lower mean values of power output. From the participant reports and the relatively crude thigh girth measurements, it would appear that the higher intake levels produced greater levels of leg pump which acted as a hindrance during high speed, high intensity cycle sprints. However, this is not to imply that the higher intake levels would be disadvantageous in all sports situations and could possibly prove to be beneficial in some particular settings. Significant increases of blood flow to exercising muscles may provide training benefits for some athletes during certain types of competition or physical conditioning. For example, the high degree of leg pump might provide unique athletic conditioning benefits to those in the competitive bodybuilding field and others during particular phases of training.

## Conclusion

Chronic supplementation of GPLC appears to provide benefits that are dose dependent. While acute supplementation of 4.5 grams was previously shown to provide significant enhancement of anaerobic work capacity, the present study suggests that chronic supplementation of GPLC at 3.0 or 4.5 grams daily does not improve anaerobic performance of repeated high speed high intensity bouts and may actually produce detrimental effects with high velocity, high intensity exercise. However, these results also suggest that 1.5 g GPLC does provide enhancement of anaerobic capacity. These findings also suggest that long term supplementation with this dosage (1.5 g/day) results in significantly lower lactate accumulation with high intensity exercise.

## Competing interests

The authors declare that they have no competing interests.

## Authors' contributions

PJ was responsible for study design, data collection, statistical analysis, and manuscript preparation. EG was responsible for data collection, input and analysis as well as manuscript preparation. All authors have read and approved the final manuscript.
